# miRNA expression profile as a potential tool for discrimination between bacterial and interstitial cystitis

**DOI:** 10.3389/fimmu.2026.1738839

**Published:** 2026-02-16

**Authors:** Dominika Peskar, Nika Kojc, Andreja Erman, Emanuela Boštjančič

**Affiliations:** 1 Institute of Cell Biology, Faculty of Medicine, University of Ljubljana, Ljubljana, Slovenia; 2 Institute of Pathology, Faculty of Medicine, University of Ljubljana, Ljubljana, Slovenia

**Keywords:** bacterial cystitis, expression analysis, inflammation, interstitial cystitis, microRNA, pathway analysis, reporter assay-validated targets, urinary bladder

## Abstract

**Introduction:**

Interstitial cystitis (IC) is an aseptic chronic bladder inflammation of unknown etiology and poorly understood pathophysiology with symptoms resembling bacterial cystitis (BC). There is limited data about the contribution of regulatory microRNAs (miRNAs) in IC. The study aimed to identify differences in miRNA expression between mouse models of IC and BC to find potential miRNAs that would distinguish between the two types of cystitis and to evaluate the use of the mouse model of IC as a tool to study the pathogenic mechanisms of IC in humans.

**Methods:**

Two mouse models were utilized: cyclophosphamide was used for induction of chronic aseptic cystitis, and uropathogenic E.coli for induction of acute bacterial cystitis. Potential regulatory miRNAs were selected based on publicly available human IC datasets and validated in mice. Quantitative PCR and RNA isolated from formalin-fixed, paraffin-embedded mouse bladder tissue were used. An enrichment analysis of the target mRNAs of the validated miRNAs was performed to suggest the differences in the possible mechanisms of inflammation between the IC and BC.

**Results:**

We observed differential expression of 20 of the 33 selected miRNAs in IC and BC compared to the control group, with 11 miRNAs showing the same trend of expression between mouse and human IC. There are 8 common reporter-assay (RA) validated targets (performed by others) of these miRNAs in mouse and human. Histopathological analysis of mouse IC and BC urinary bladders, miRNA expression analysis, and expression of their validated targets revealed significant differences between the urinary bladders of BC and IC mouse models. We identified *miR-301a-3p* as a possible marker of discrimination between two types of cystitis and its target gene, nuclear factor-κB (NF-κB) suppressing factor (*NKRF*).

**Conclusion:**

Our results show that miRNA expression and its RA-validated targets, and enriched signaling pathways, differ between the two types of cystitis and might depend on the type of bladder inflammation. The mouse model of IC has some similarities with human IC, confirming that it is a useful tool to identify novel potentially discriminatory biomarkers between BC and IC, such as *miR-301a-3p*, and also potential therapeutic targets for IC (e.g., *NKRF* and NF-κB).

## 1 Introduction

Interstitial cystitis (IC) is an aseptic chronic bladder inflammation of unknown etiology and poorly understood pathophysiology characterized by disturbances in the physiological micturition cycle and persistent pelvic pain (1). For undetermined reasons, women are more commonly affected by IC than men (2, 3). The clinical presentation of IC is uncharacteristic. Patients present an increase in micturition frequency, reduced volume of voided urine, painful urination, nocturia, and hematuria, which overlap with symptoms of other lower urinary tract disorders (LUTDs) (1, 4). One of the LUTDs that share symptoms similar to IC is urinary tract infection (UTI), which is one of the most often diagnosed bacterial infections (5). Women are more predisposed to UTI than men, and in more than 80% of cases, UTI is caused by uropathogenic Escherichia coli (UPEC) (5). Although antibiotic treatment eliminates UTI-caused symptoms, it is estimated that symptoms recur in more than 20% of treated UTIs (6).

Along with clinical symptoms, UTI is often defined as the presence of more than 1000 colony-forming units of bacteria in the urine (7). However, in 20% of patients with clinical signs of UTI, the urine may be sterile (7, 8). In these cases, together with the high frequency of recurrence of urinary symptoms, a UTI can hardly be distinguished clinically from an IC. Since the diagnosis of IC is based on the exclusion of other LUTDs with similar symptoms, biomarkers that enable discrimination between two LUTDs are in high demand.

MicroRNAs (miRNAs) are small non-coding RNA molecules consisting of about 22 nucleotides that can regulate gene expression. A single miRNA can bind to many different target mRNAs and thus prevent their translation into protein (9). Their expression is highly time- and tissue-specific, and deviations in their expression are often linked to the development of various pathologies (10). Additionally, their high stability in different types of tissue samples, including samples of lower quality, makes them attractive diagnostic and therapeutic targets (11, 12).

In the present study, a mouse model of IC was used as a model of chronic bladder inflammation, and a mouse model of bacterial cystitis (BC) was used as a model of acute bladder inflammation. There were two aims of the study. First, to identify differences in miRNA expression profiles in the urinary bladders of these two mouse models to determine miRNAs as potential differential biomarkers between IC and BC. Second, to assess the use of the mouse model of IC as a tool to investigate potential pathogenic mechanisms of IC in humans. For the second purpose, potential target miRNAs were selected based on publicly available datasets obtained in human patients with IC and validated in mice. For miRNA validation, formalin-fixed, paraffin-embedded (FFPE) mouse bladder tissue samples were obtained from the archive, further emphasizing the high value of long-stored tissue samples in an effort to reduce the number of laboratory animals used in preclinical studies. Additionally, enrichment analysis of target mRNAs of the selected and validated miRNAs was performed to elucidate the possible differences in inflammatory mechanisms between the IC and BC models.

## 2 Materials and methods

**Figure 1:**
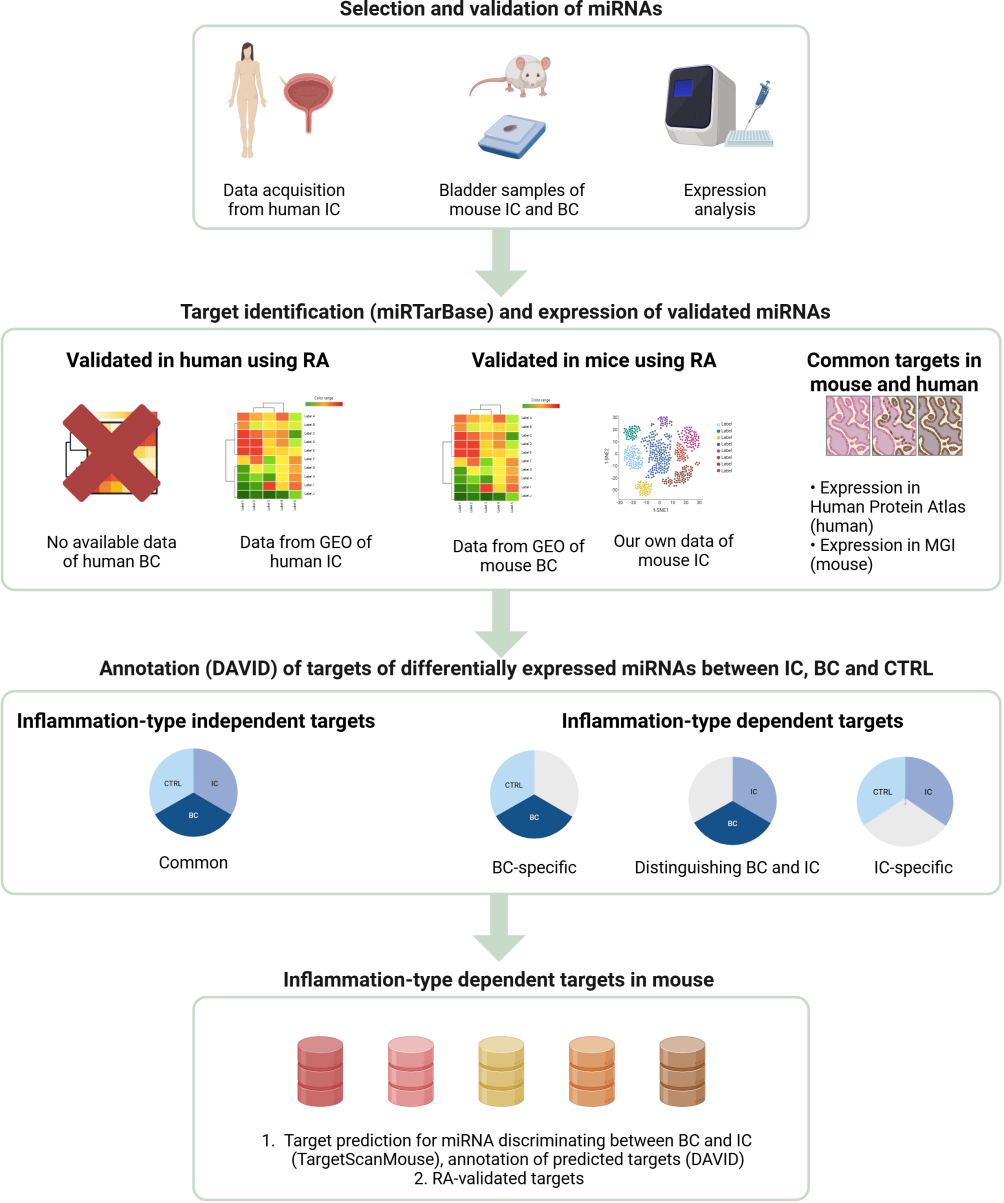
Flow chart of miRNA screening. IC, interstitial cystitis; BC, bacterial cystitis; CTRL, control; GEO, Gene Expression Omnibus; RA, reporter assay. Created with BioRender.com.

A schematic representation of the workflow is presented in **Figure 1** to make it easier to follow the sequence of methods.

### 2.1 Animal models

To minimize the number of experimental animals used, archived FFPE samples of mouse urinary bladders obtained from our previous experiments were used. The model of IC was established in adult C57BL/6J mice as previously described (13). Briefly, IC was established by i.p. administration of cyclophosphamide (80 mg/kg body weight) four times (on days 0, 2, 4, and 6) in the treated group of animals, while the control group (CTRL) received the same amount of sterile saline at the same time points. The animals were euthanized by CO_2_ asphyxia on day 8 of the experiment. The bladders were excised and immediately fixed overnight in 10% buffered formalin. The archived FFPE bladder samples from 6 female mice with IC and 3 female CTRL mice were included in the study.

The model of BC was established in adult female C57BL/6J mice as previously described (14). Briefly, UPEC bacteria (strain UTI89) were used for the induction of acute bacterial cystitis. Mice were inoculated under anesthesia with a bacterial suspension (10^7^ CFU) via transurethral catheterization using a flexible catheter (Intramedic, Becton Dickinson, USA) sheathed over a needle connected to a syringe. The mice were euthanized 24 hours after infection and the excised urinary bladders were fixed in 10% formalin overnight. The archived FFPE urinary bladder samples from 5 female mice with BC were included in the study.

### 2.2 Histopathological evaluation of urinary bladder inflammation

FFPE bladder samples from experimental animals were cut into 4 μm thick slices using a microtome, then deparaffinized and stained with hematoxylin and eosin according to the standard protocol. To ensure the impartiality of the histopathologic evaluation, the samples and histological slides were coded until the end of the evaluation of all bladder samples. The histological slides were scanned with an optical scanner (NanoZoomer S360 Hamamatsu Photonics K.K., Japan).

The specimens (three per animal) were examined with a light microscope (Eclipse E600 Nikon, Japan) at 600x magnification. At the same time, the scanned slides were analyzed on a computer with an optical reader (NanoZoomer S360 Hamamatsu Photonics K.K., Japan) at 400x magnification.

For each sample, the total area of the lamina propria (LP; excluding the urothelium and muscle layer) was measured using NDP.view2 (version 3.3.26, Hamamatsu Photonics K.K., Hamamatsu, Japan), and the result was expressed in mm². Immune cells and small blood vessels were counted in the entire LP (vessels with no more than 2 layers of smooth muscle cells were included), and the results were expressed as the number of inflammatory cells per mm^2^, neutrophils per mm^2^ and blood vessels per mm^2^. All measurements were performed by the same pathologist who was blinded to the treatment of the animals.

### 2.3 Datasets and miRNA selection

Four publicly available datasets of high-throughput analytical data were used in the study (**Table 1**). Two of the datasets were obtained from bladder tissue biopsies from patients with IC (15, Arai et al. (16)). The data of interest from these two studies were fully available as **Supplementary Material** of the published studies. One of the datasets was obtained from bladder tissue samples of a mouse model of BC (17). The data from this study were accessed from the NCBI GEO platform (accession number GSE72007). Differential expression analysis of this data was performed using the GEO2R web tool. One of the datasets was from bladder tissue samples from a mouse model of IC obtained in one of our previous studies (13) and available on the NCBI GEO platform (accession number GSE221783). No data on miRNA expression in human bladders with bacterial infection were available at the time of preparing the publication.

**Table 1:** Datasets utilized in the present study.

Reference	Samples	Method	Data of interest
Gheinani et al. (15)	Human, bladder biopsy samples, IC without Hunner lesions (n=6, female) and CTRL (n=6, 4 male and 2 female)	RNA sequencing (Illumina HiSeq 2000 platform)	miRNA (41 significantly downregulated, 21 significantly upregulated in IC compared to CTRL), mRNA
Arai et al. (16)	Human, bladder biopsy samples, IC with Hunner lesions (n=5, female), IC without Hunner lesions (n=3, female), CTRL (n=5, male)	RNA sequencing (Illumina HiSeq 2000 platform)	miRNA (203 significantly downregulated, 163 significantly upregulated in IC compared to CTRL)
Peskar et al. (13)	C57BL/6J mice, whole bladder samples, IC induced with 4 applications of cyclophosphamide (n=3, female), CTRL (n=3, female)	RNA sequencing (Illumina NovaSeq 6000 platform)	mRNA
Spencer et al. (17)	C57BL/6 mice, whole bladder samples, BC induced with intravesical inoculation of UTI89 (n=4, female, 48h after inoculation), CTRL (n=4, female)	Microarray (SurePrint G3 Mouse GE 8x60K)	mRNA

BC, bacterial cystitis; CTRL, control; IC, interstitial cystitis.

First, a list of miRNAs with significantly deregulated expression (p<0.05 relative to corresponding controls) in bladders of patients with IC was constructed using two datasets (15, 16) (**Table 1**). All significantly deregulated miRNAs from the Gheinani et al. dataset (15) (n=62) and 20 most significantly upregulated and 20 most significantly downregulated miRNAs from the Arai et al. (16) dataset were added to the initial list. After excluding duplicates, the list contained 98 miRNAs with significantly deregulated expression in patients with IC compared to CTRL.

Next, the miRNA list was filtered based on the expression of their targets in mouse bladders. For this purpose, mRNA targets of the selected miRNAs were pooled from the TarBase v.8 Karagkouni et al. (18). Only validated targets of each miRNA (validated by qPCR, Western blot, or reporter assay (RA) in human tissue) were pooled. The expression of mouse targets corresponding to the pooled human targets was then inspected in mouse bladders using the available mRNA sequencing dataset from Peskar et al. (13) (**Table 1**). The miRNAs with targets not found in mouse bladders were excluded, and 33 miRNAs were selected for further analysis.

### 2.4 RNA isolation, reverse-transcription and quantitative polymerase chain reaction

#### 2.4.1 RNA isolation

From each FFPE tissue block, 3-4 10-μm-thick sections were cut and placed in sterile 2.0-ml microcentrifuge tubes. RNA isolation was performed semi-automatically using the Maxwell RSC (Promega, Madison, WI 53711-5399, USA) and a combination of the Maxwell^®^ RSC RNA FFPE kit (Promega, Madison, WI 53711-5399, USA; code AS1440) and the Maxwell^®^ RSC miRNA tissue kit (Promega, Madison, WI 53711-5399, USA; code AS1460). This combination enables the extraction of miRNAs from FFPE tissue samples, as suggested by the manufacturer and published by Azzalini et al. (19). In detail, the tissue was deparaffinized with 300 µL of mineral oil, followed by overnight protein digestion and the DNase digestion step as recommended in the Maxwell^®^ RSC RNA FFPE kit instructions. Thioglycerol homogenization solution (enhancer solution) and lysis buffer were then added to the aqueous solution of the digested samples according to the manufacturer’s procedures. The entire volume was then transferred to the cartridge of the Maxwell^®^ RSC miRNA Tissue Kit to allow isolation of total RNA, including miRNAs, but without an additional DNase digestion step. Elution of samples was performed in 50 µL of nuclease-free water (Promega, Madison, WI 53711-5399, USA). RNA was quantified using the NanoDrop-1000 and the Qubit fluorimeter using the HS RNA quantification kit.

#### 2.4.2 Reverse transcription

The isolated RNA was reverse transcribed (RT) into cDNA using the miRCURY LNA RT Kit (Qiagen; Hilden, Germany) according to the manufacturer’s protocol. The successful RT procedure was confirmed by the addition of spike-in RNA (UniSp6) and its subsequent quantification. Ten µL of reaction master mix contained 2 µL of miRCURY RT Reaction Buffer 5x, 1 µL of miRCURY RT Enzyme Mix 10x, 0.5 µL of UniSp6 Spike-in with 6.5 µL of total RNA (10 ng). The reaction was run at 42°C for 60 minutes followed by incubation at 95°C for 5 minutes (heat inactivation of reverse transcriptase) and immediate cooling to 4°C.

#### 2.4.3 PCR efficiency estimation

SYBR Green was used to measure the expression levels of selected miRNAs. Prior to qPCR, efficiency was determined using three pools of RNA samples obtained from IC, BC and CTRL samples. After RT, the cDNA of miRNA was diluted in four steps, ranging from a 4-point dilution to a 256-point dilution, and primers were tested for qPCR efficiency. All qPCR efficiency reactions were performed on a QuantStudio 7Pro (Thermo Fisher Scientific) in triplicates.

#### 2.4.4 Quantitative polymerase chain reaction

For quantification using qPCR, a pre-designed mixture of primers specific for miRNAs expression was used. A miRCURY LNA SYBR GREEN Kit was used for the analysis of selected miRNAs (summarized in **Table 2**), relative to the geometric mean of the expression of reference miRNAs proposed by the manufacturer (*miR-103a-3p* and *miR-191*). Briefly, 3 µL of diluted (1:60) cDNA was used in a 10 µL reaction volume with 1 µL of miRCURY LNA miRNA PCR Primer Assay 10x, 5µL of MasterMix, and 1µL of PCR-grade H_2_O. Amplification was performed as follows: 95°C for 2 min, 40 cycles of denaturation at 95°C for 10 s and annealing at 56°C for 1 min, followed by melting curve analysis, on a QuantStudio 7Pro (Thermo Fisher Scientific). All qPCR reactions were performed in duplicates.

**Table 2:** The list of the analyzed miRNAs, including reference miRNAs and their specification (catalogue) numbers.

miRNAs	Cat. number	miRNAs	Cat. number	miRNAs	Cat. number
*hsa-let-7b-5p*	YP00204750	*hsa-miR-107*	YP00204468	*hsa-miR-204-5p*	YP00206072
*hsa-let-7c-5p*	YP00204767	*hsa-miR-126-3p*	YP00204227	*hsa-miR-206*	YP00206073
*hsa-let-7e-5p*	YP00205711	*hsa-miR-126-5p*	YP00206010	*hsa-miR-301a-3p*	YP00205601
*hsa-let-7f-2-3p*	Different from mm	*hsa-miR-129-5p*	YP00204534	*hsa-miR-320a*	YP00206042
*hsa-let-7f-5p*	YP00204359	*hsa-miR-133b*	YP00206058	*hsa-miR-326*	YP00204512
*hsa-miR-10b-5p*	YP00205637	*hsa-miR-140-3p*	YP00204304	*hsa-miR-375-3p*	YP00204362
*hsa-miR-19a-3p*	YP00205862	*hsa-miR-141-3p*	YP00204504	*hsa-miR-381-3p*	YP00205887
*hsa-miR-20b-5p*	YP00204755	*hsa-miR-145-5p*	YP00204483	*hsa-miR-424-5p*	Absent in mm
*hsa-miR-29c-3p*	YP00204729	*hsa-miR-146a-5p*	YP00204688	*hsa-miR-495-3p*	YP00206015
*hsa-miR-32-5p*	YP00204792	*hsa-miR-151a-5p*	Questionable.	*UniSp6*	YP00203954
*hsa-miR-34a-5p*	YP00204486	*hsa-miR-190a-5p*	YP00204763	*hsa-miR-191-5p*	YP00204306
*hsa-miR-96-5p*	YP00204417	*hsa-miR-200c-3p*	YP00204482	*hsa-miR-103a-3p*	YP00204063

Cat., catalogue; mm, Mus musculus.

The quality of miRNA analysis was checked by amplifying UniSp6 (to test the equality of RT between the samples) and by amplifying reference miRNAs (*miR-103a-3p* and *miR-191*) and calculate their geometric mean (to test the equality of generated Cqs for each miRNAs and calculated geometric mean between the samples).

### 2.5 Statistical analysis

Results were presented as relative gene expression using the ΔCq. All Cq values were corrected for PCR efficiencies, and to obtain ΔCq, the geometric mean of the Cq values of the reference genes (Cq_RG_) was deducted from the Cq values of the gene of interest (GOI, Cq_GOI_). For comparison of miRNA expression differences between independent sample groups (*e.g.*, IC vs. BC, etc.), ΔCq and the Mann-Whitney test were used. Statistical analysis of the data was performed using SPSS version 27 (SPSS Inc., Chicago, IL, USA). Differences were considered significant at p-values < 0.05.

### 2.6 Target gene identification and enrichment analysis

To identify miRNA targets, we searched miRTarBase (20) for validated targets of statistically differentially expressed miRNAs separately for human and mouse miRNAs. In the first step, we searched for the expression of RA-validated targets using publicly available datasets and our own data (**Table 1**). Next, we searched for common targets in humans and mice. Their expression, location, and function were obtained from the Human Protein Atlas (21) and Mouse Genome Informatics (22) for human and mouse, respectively. In the second step, we used the DAVID tool (23) for functional annotation and enrichment analysis of the targets of differentially expressed miRNAs between BC, IC, and CTRL. In the final step, we searched for common validated targets (TargetScan 8.0) Karagkouni et al. (18) and performed target enrichment analysis for IC-specific miRNAs (**Figure 1**). The figures depicting the results of the enrichment analysis were constructed using the online tool SRplot (24).

## 3 Results

### 3.1 Detection of selected miRNAs in mouse bladders and differential expression between mouse groups

As described in the Methods section, 33 miRNAs were selected for confirmatory analysis of expression in mouse FFPE samples. Based on the miRNA nucleotide sequence homology between human and mouse and the availability of primers for the detection of miRNA, the expression of 30 miRNAs was analyzed by qPCR in the mouse bladder (listed in **Table 2**). Only two of the miRNAs analyzed were not sequence homologues between Mus musculus and Homo sapiens.

**Figure 2:**
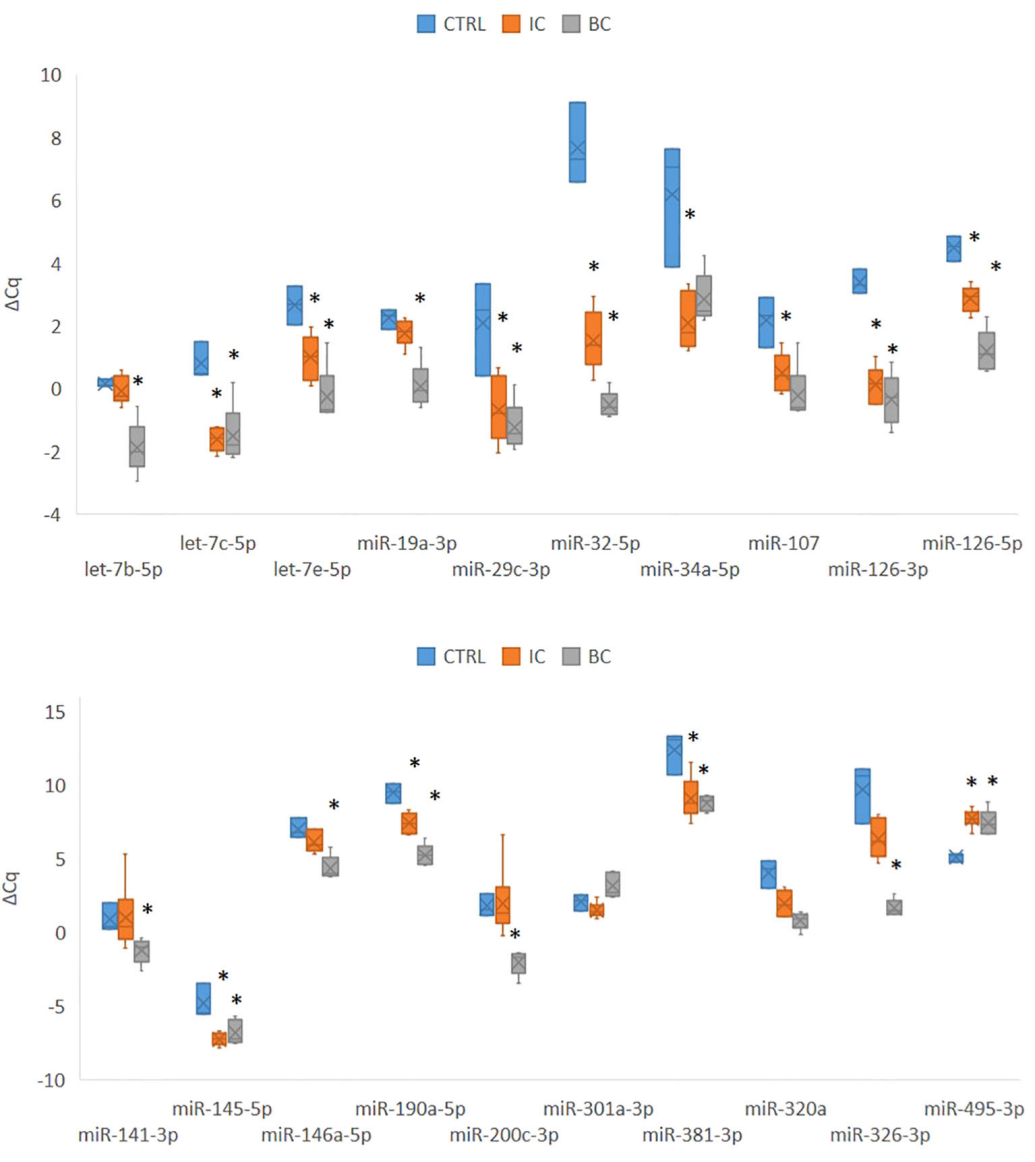
Expression of 20 significantly deregulated selected miRNAs in all three experimental animal groups. p-value was calculated using Mann-Whitney test. Legend: *p < 0.05. BC, bacterial cystitis; CTRL, control; IC, interstitial cystitis.

Of the 30 miRNAs analyzed, 4 miRNAs were not expressed above the detection limit by qPCR (*miR-20b, miR-129-5p, miR-206, miR-375*). The qPCR analysis was therefore continued for the remaining 26 miRNAs. However, for two miRNAs (*miR-381, miR-204-5p*), the PCR efficiency was difficult to calculate; therefore, more careful analysis of the results was required. Of the 26 miRNAs analyzed, 20 miRNAs were significantly differentially expressed between at least two experimental animal groups (i.e. CTRL vs. IC, CTRL vs. BC or BC vs. CTRL) (**Figure 2**). Generally, the expression of the analyzed miRNAs was upregulated in the IC and BC groups relative to the CTRL groups, with the exception of *miR-495-3p*. Six miRNAs showed no statistically significant difference in expression between the animal groups: *let-7f-5p, miR-10b-5p, miR-96-5p, miR-133b, miR-140-3p*, and *miR-204-5p*.

Of the 20 differentially expressed miRNAs, 12 miRNAs were significantly deregulated in mouse bladders with IC compared to controls; 17 of the analyzed miRNAs were significantly deregulated in mouse bladders with BC compared to controls; 12 miRNAs were significantly deregulated in mouse bladders with IC compared to BC. The differential expression of the individual miRNA in each comparison is summarized in **Table 3**.

**Table 3:** The list of miRNAs analyzed in the bladders of CTRL, IC and BC mice.

miRNA sub-group	miRNA	IC vs. CTRL	BC vs. CTRL	IC vs. BC	miRNA	IC vs. CTRL	BC vs. CTRL	IC vs. BC	miRNA sub-group
Inflammation type-independent	*let-7c-5p*	p = 0.020	p = 0.025	ns	*miR-34a-5p*	p = 0.020	ns	ns	**Inflammation type-dependent**
	*miR-126-3p*	p = 0.020	p = 0.025	ns	*miR-107*	p = 0.039	ns	ns	
	*miR-145-5p*	p = 0.020	p = 0.025	ns	*let-7b-5p*	ns	p = 0.025	p = 0.011	
	*miR-29c-3p*	p = 0.039	p = 0.025	ns	*miR-141-3p*	ns	p = 0.025	p = 0.020	
	*miR-495-3p*	p = 0.020	p = 0.025	ns	*miR-146a-5p*	ns	p = 0.025	p = 0.018	
	*let-7e-5p*	p = 0.020	p = 0.025	p = 0.045	*miR-19a-3p*	ns	p = 0.025	p = 0.011	
	*miR-126-5p*	p = 0.020	p = 0.025	p = 0.011	*miR-200c-3p*	ns	p = 0.025	p = 0.006	
	*miR-190a-5p*	p = 0.020	p = 0.025	p = 0.006	*miR-326-3p*	ns	p = 0.025	p = 0.006	
	*miR-320a*	p = 0.039	p = 0.025	p = 0.045	*miR-381-3p*	ns	p = 0.025	ns	
	*miR-32-5p*	p = 0.020	p = 0.025	p = 0.006	*miR-301a-3p*	ns	ns	p = 0.006	
Non-significant	*let-7f-5p*	ns	ns	ns	*miR-204-5p*	ns	ns	ns	**Non - significant**
	*miR-10b-5p*	ns	ns	ns	*miR-133b*	ns	ns	ns	
	*miR-140-3p*	ns	ns	ns	*miR-96-5p*	ns	ns	ns	

Statistical significance for each group comparison is presented. p < 0.05 was considered as statistically significant; ns: not significant. BC, bacterial cystitis; CTRL, control group; IC, interstitial cystitis.

### 3.2 Comparison of miRNA expression and their validated targets between experimental IC and human IC data

Only two miRNAs were significantly deregulated in both patient subsets (*miR-126-5p* and *miR-141-3p*). A similar trend of regulation between mouse IC and human IC datasets was found for the following miRNAs: *miR-10b-5p, miR-19a-3p, miR-126-3p, miR-126-5p, miR-141-3p, miR-301a-3p, miR-381-3p* compared to ulcerative IC, and for *miR-133b, miR-204-5p, miR-145-5p*, and *miR-326-3p* compared to non-ulcerative IC. The results are summarized in **Table 4**.

**Table 4:** Summary of trends in the deregulation of miRNAs analyzed in the bladders of mice with experimentally induced IC compared to their deregulation in the bladders of patients with non-ulcerative and ulcerative IC.

miRNA	Homology mmu - hsa	Deregulation in mouse IC	Deregulation in non-ulcerative human IC	Deregulation in ulcerative human IC
***miR-133b***	✓	**↑**	**↑**	
***miR-204-5p***	✓	**↑**	**↑**	
***miR-301a-3p***	✓	**↑**		**↑**
***miR-381-3p***	✓	**↑**		**↑**
*miR-320a*	x	**↑**		**↓**
***miR-326-3p***	x	**↑**	**↑**	
*let-7c-5p*	✓	**↑**		**↓**
***miR-145-5p***	✓	**↑**	**↑**	
*let-7e-5p*	✓	**↑**		**↓**
*let-7f-5p*	✓	**↑**		**↓**
*miR-29c-3p*	✓	**↑**	**↓**	
*miR-34a-5p*	✓	**↑**	**↓**	
***miR-126-5p***	✓	**↑**	**↓**	**↑**
***miR-141-3p***	✓	**↑**	**↓**	**↑**
*miR-146a-5p*	✓	**↑**	**↓**	
*miR-200c-3p*	✓	**↑**	**↓**	
*miR-495-3p*	✓	**↓**	**↑**	
*let-7b-5p*	✓	**↑**		**↓**
***miR-10b-5p***	✓	**↑**		**↑**
***miR-19a-3p***	✓	**↑**		**↑**
*miR-32-5p*	✓	**↑**	**↓**	
*miR-96-5p*	✓	**↑**	**↓**	
*miR-140-3p*	✓	**↑**		**↓**
*miR-107*	✓	**↑**		**↓**
***miR-126-3p***	✓	**↑**		**↑**
*miR-190a-5p*	✓	**↑**	**↓**	

mmu, Mus musculus; hsa, Homo sapiens; IC, interstitial cystitis. The miRNAs marked in bold showed a similar trend of deregulation between experimental IC and one of the human datasets. Empty squares indicate that the respective miRNA was not significantly deregulated in the bladders of patients in a subgroup.

Using miRTarBase (20), we searched for the RA-validated target mRNAs for all 20 significantly deregulated miRNAs in both mice (mmu) and humans (hsa) (**Supplementary Tables S1**, **S2**) for data from humans and mice, respectively). Of the 26 miRNAs analyzed in the mouse bladders, 12 were significantly deregulated between the IC and CTRL groups. We found RA-validated targets for all 12 miRNAs in the human miRTar database (a total of 487 targets, 401 of which were unique and expressed in human bladders) and for 8 out of 12 miRNAs in the mouse miRTarBase (a total of 41 targets, all of which were unique and expressed in mouse bladders). No RA-validated targets were listed for *mmu-miR-126-3p, mmu-miR-126-5p, mmu-miR-145-5p* and *mmu*-*miR-190a-5p*. The results of bladder-specific expression of the target mRNAs in human and mouse bladders are summarized in **Supplementary Table S3**.

**Figure 3:**
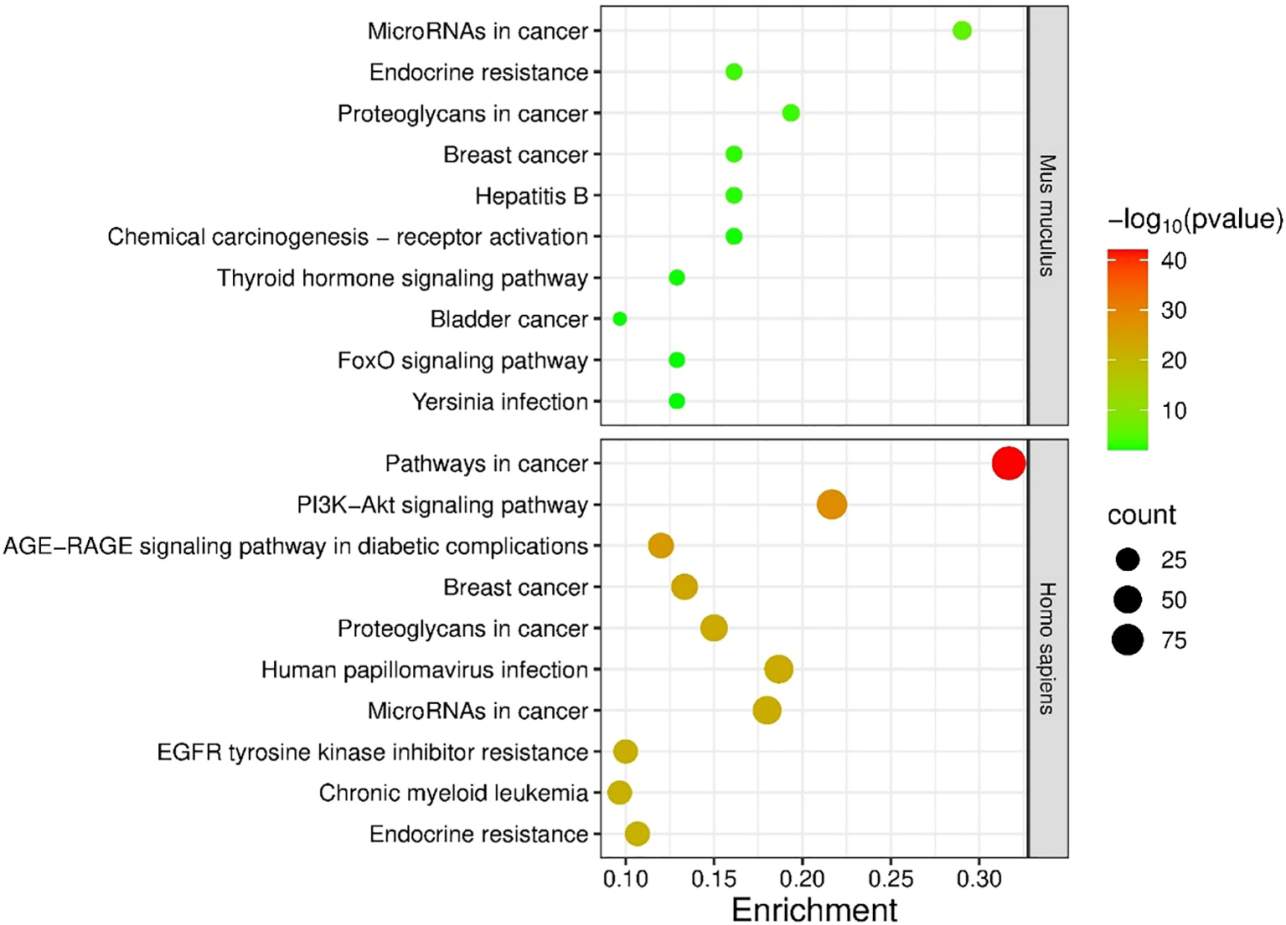
Enrichment analysis of targets of miRNAs expressed in the urinary bladders with differential expression between IC and CTRL. Shown are 10 KEGG pathways that are most significantly enriched in human (Homo sapiens) and mouse (Mus musculus). p-values are represented by the bubble color and gene counts are represented by the bubble size. p-value < 0.05 was considered statistically significant. BC, bacterial cystitis; IC, inetrstitial cystitis.

Functional annotation of the unique bladder-expressed targets (401 in human and 41 in mouse) showed enrichment of mouse genes in 38 KEGG pathways, and enrichment of human genes in 156 KEGG pathways (**Supplementary Table S3**). Interestingly, mouse and human shared all but one KEGG pathway (namely the ‘Phagosome’ pathway). Thirty-one and 149 of the pathways were significantly enriched in mouse and human, respectively. Of the 10 most enriched pathways, 4 were enriched in both species: ‘MicroRNAs in cancer’, Endocrine resistance’, ‘Proteoglycans in cancer’, and ‘Breast cancer’). The top 10 most enriched pathways are presented in **Figure 3**.

Only 8 of the targets regulated by the same miRNAs were the same in mice and humans (*MYC, AKT3, EIF3J, SNHG12, HSPB6, NOTCH1, SIRT1*, and *DLL1*). For these targets, data on cell and tissue expression, function, and sublocalization were derived from the Human Protein Atlas (HPA) for human targets and from MGI for mouse targets where available (**Supplementary Table S3**). The results of the search show that the functional role of the genes was largely preserved and that the cellular sublocalization of the proteins was highly concordant between both species. Most of the targets were highly expressed in the urinary bladder of both species. Interestingly, most of the genes were detected in the majority of immune cells. Some genes (*HSPB6* and *DLL1*) displayed more species-specific expression patterns. Additionally, we searched for expression data of these common targets in the human IC and mouse IC datasets, which are listed in **Table 1**. Of the 8 genes, only *NOTCH1* was significantly deregulated in patients with IC, while none of the genes were differentially expressed in mouse IC. The data is summarized in **Supplementary Table S3**.

### 3.3 Comparison of bladder inflammation in BC and IC mouse models

#### 3.3.1 Different histopathological features of the bladder wall depending on the type of inflammation - acute or chronic

**Figure 4:**
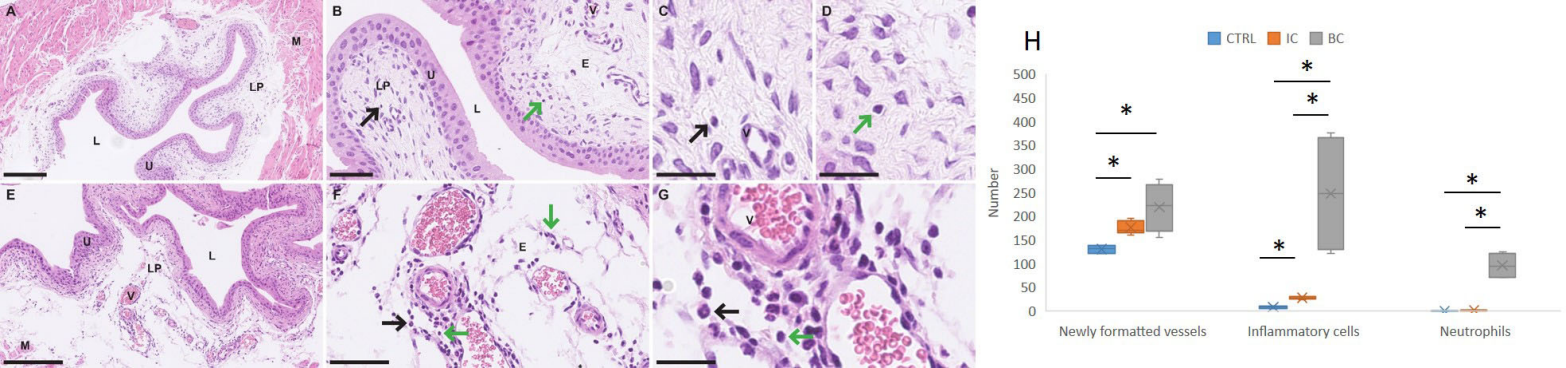
Differences in the urinary bladder wall of mice with IC and BC at the histological level. **(A–D)** Representative micrographs of the urinary bladder wall of mice with IC (H&E staining). **(E–G)** Representative micrographs of the urinary bladder wall of mice with BC (H&E staining). **(H)** Graphical representation of the results of the quantitative analysis showing the differences in the number of blood vessels, all extravasal immune cells and neutrophils in the lamina propria of the urinary bladder. Legend: E, edema; L, lumen of the urinary bladder; LP, lamina propria; M, muscle; V, blood vessel; black arrows, immune cells; green arrows, neutrophilic granulocytes. *p-value was calculated using Mann-Whitney test and p < 0.05 was considered statistically significant; resolution of figures is 250 μm for **(A, E)**; 50 μm for B and F; 25 μm for **(C, D, G)**. BC, bacterial cystitis; IC, interstitial cystitis.

Inflammatory changes in the lamina propria (LP) of the bladder were assessed histopathologically by the number of inflammatory cells, including neutrophils infiltrating the LP, and the number of newly formed small blood vessels. Based on the shape of nuclei, we differentiated between neutrophils and mononuclear immune cells (lymphocytes and macrophages). In the bladder wall of mice with chronic bladder inflammation (IC), the edema of the LP was mild, and there were scattered inflammatory cells with very few neutrophils among them (**Figures 4A-D**). In the LP of mouse bladders with acute inflammation (BC), there was marked edema and clusters of neutrophils were arranged in small vessels, extravascularly around small vessels, and throughout the LP. There were also clusters of mononuclear immune cells in LP (**Figures 4E-G**).

Quantitative histopathological analysis revealed a significant increase in the number of inflammatory cells in the LP of the bladder wall of animals with IC and BC compared to control animals (p = 0.025 and p = 0.034, respectively). This analysis, in addition, revealed a significant increase in *de novo* -formed vessels in the LP of the bladder of animals with IC and BC compared to control animals (p = 0.025 and p = 0.034, respectively). We also observed a statistically significantly elevated number of neutrophils in mice with BC compared to control mice (p = 0.028), whereas this number was not significantly elevated in animals with IC compared to controls. Furthermore, we also observed a significant increase in the number of all inflammatory cells, including neutrophils in the LP of mice with BC compared to the LP of mice with IC (p = 0.016) (**Figure 4H**).

#### 3.3.2 Differential expression of miRNAs depending on the type of bladder inflammation

We categorized the selected miRNAs into either i) inflammation type-independent miRNAs or ii) inflammation type-dependent miRNAs based on their expression in the individual animal groups. The category of inflammation type-independent miRNAs included those that were significantly deregulated in both the IC and BC groups compared to controls (n=10; **Table 3**). Five of these miRNAs were also significantly deregulated between the IC and BC groups, which was attributed to the differences in the extent of inflammatory processes, as all miRNAs showed the same direction of deregulation (up or down) compared to the CTRLs. On the other hand, the category of inflammation type-dependent miRNAs included those that were significantly deregulated only in IC compared to CTRL (n=2), only in IC compared to BC (n=7), or only in BC compared to CTRL (n=7; **Table 3**). Six of the seven miRNAs that were deregulated between BC and CTRL were also significantly deregulated between IC and BC. Therefore, *miR-301a-3p* was the only miRNA that was exclusively significantly deregulated in IC compared to BC and also the only one with inverse regulation between these two types of bladder inflammation (**Figure 2**, **Table 3**).

#### 3.3.3 Identification of RA-validated targets of inflammation type-related miRNAs

Furthermore, we used miRTarBase to search for the RA-validated targets (determined in mouse tissue) of inflammation type-related miRNAs (n=10). Subsequently, expression data for individual targets in mouse urinary bladder were obtained from the literature (17) and from our own data (13) for BC and IC, respectively.

For inflammation type-dependent miRNAs that were significantly deregulated exclusively between IC and CTRL groups (*miR-34a-5p, miR-107*; n=2), 12 RA-validated targets were identified, of which 3 were significantly deregulated in the BC and only 1 in the IC model. For 1 miRNA (*miR-381-3p*) that was only significantly deregulated between BC and CTRL animal groups, only 1 RA-validated target was found that was not significantly deregulated in any of the models. For 1 miRNA (*miR-301a-3p*) with significant deregulation exclusively between IC and BC groups, 4 targets were found, 1 of which was significantly deregulated in the BC model. For miRNAs with combined significant deregulation in BC vs CTRL and IC vs BC (*let-7b-5p, miR-141-3p, miR-146a-5p, miR-19a-3p, miR-200c-3p, miR-326-3*p; n=6), 44 RA-validated targets were identified. Of these, 13 targets were significantly deregulated in BC, while only one target gene was significantly deregulated in IC urinary bladders compared to the corresponding controls.

**Figure 5:**
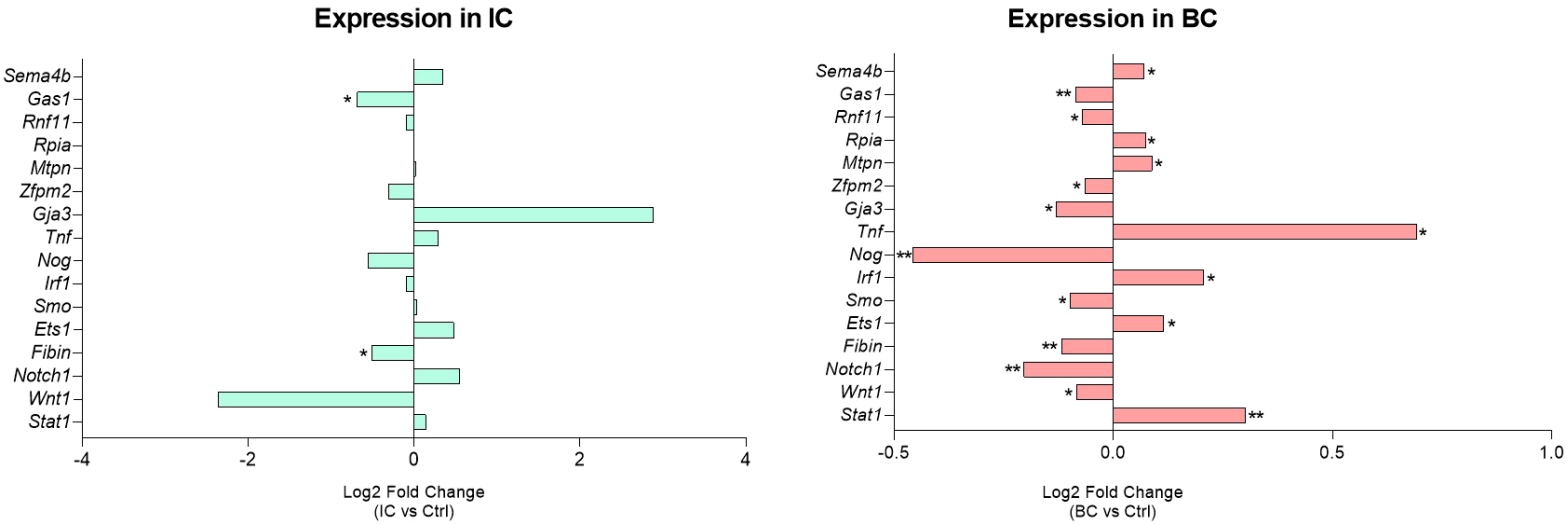
Urinary bladder expression of RA-validated targets of inflammation type-dependent miRNAs. Log2 FC relative to the corresponding controls of the significantly deregulated targets are presented. Legend: *p < 0.05, **p < 0.01. BC, bacterial cystitis; IC, interstitial cystitis.

Twelve of the 16 significantly deregulated targets had the same direction of deregulation, while 4 targets showed reverse regulation between the IC and BC animal groups, namely *Notch1, Gja3, Smo*, and *Irf1*. The change in expression of the 16 significantly deregulated targets (each individually compared to the control) is summarized in **Figure 5**. Additionally, the data of all RA-validated targets of inflammation type-dependent and -independent miRNAs are summarized in **Supplementary Table S4**.

#### 3.3.4 Annotations of RA-validated targets of miRNAs based on the inflammation type-dependency

As described above, the differentially expressed miRNAs were categorized into inflammation type-dependent and inflammation type-independent groups. We used the functional annotation tool (DAVID) to analyze the differences in KEGG pathway enrichment between the RA-validated targets of inflammation type-dependent (n=58) and inflammation type-independent (n=29) miRNAs.

**Figure 6:**
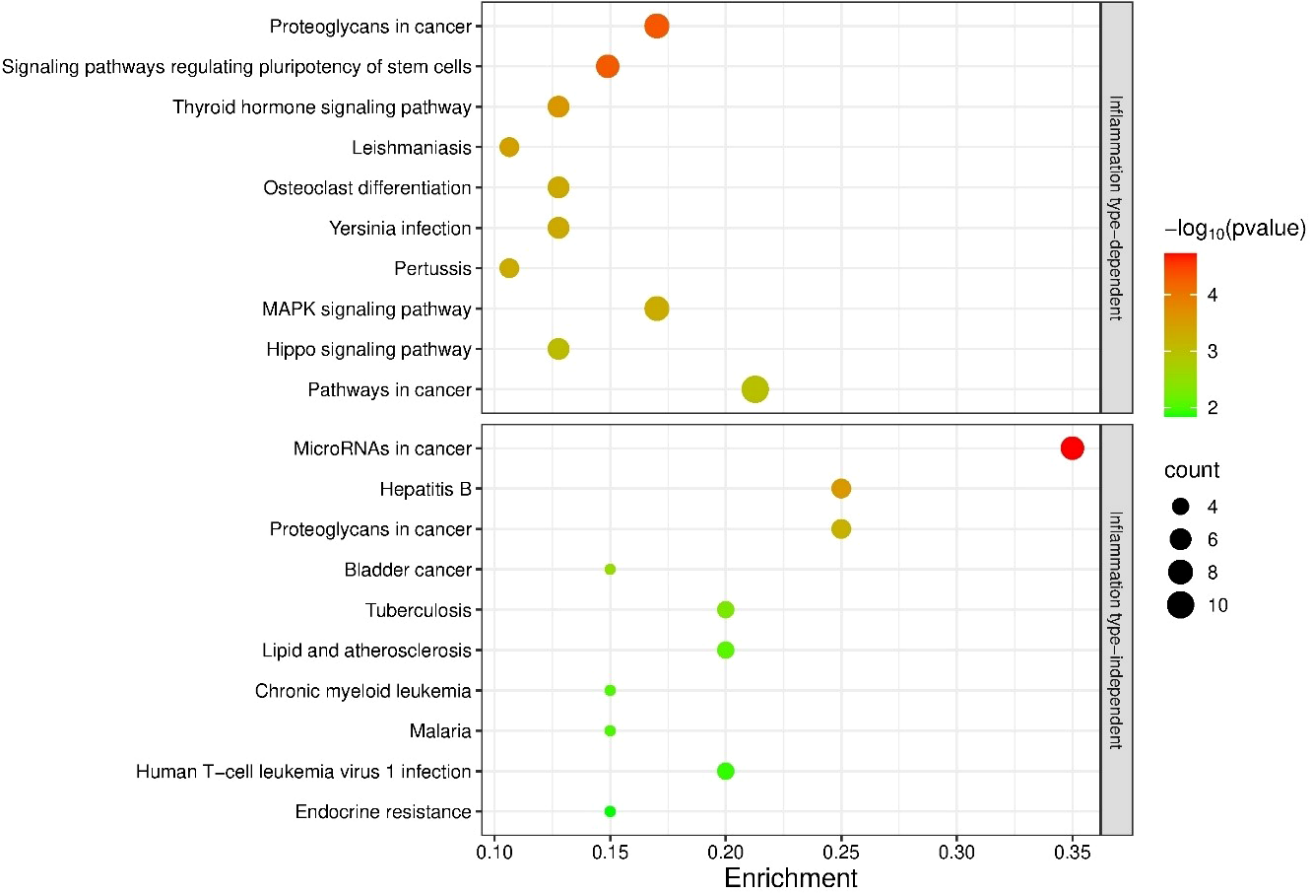
Enrichment analysis of the targets of bladder inflammation type-dependent and inflammation type-independent miRNAs. The 10 most significantly enriched KEGG pathways are shown. p-values are represented by the bubble color and gene counts are represented by the bubble size. p-value < 0.05 was considered statistically significant.

Targets of inflammation type-dependent miRNAs were enriched in 53 KEGG pathways, 40 of which were significantly enriched. The targets of inflammation type-independent miRNAs were enriched in 36 KEGG pathways, of which 24 were significantly enriched. Of the 70 unique pathways, 19 KEGG pathways were shared between subgroups (36% for the type-dependent and 53% for the type-independent; **Supplementary Table S4**). The 10 most enriched KEGG pathways are shown in **Figure 6**. Only 1 of the top 10 pathways was shared between the type-dependent and type-independent groups (‘Proteoglycans in cancer’).

#### 3.3.5 Annotations of RA-validated targets of *miR-301a-3p*

As mentioned above, *miR-301a-3p* was the only miRNA that was differentially and inversely expressed exclusively between IC and BC (upregulated in IC, downregulated in BC). Based on the miRTarBase data, 4 targets of this miRNA were RA-validated in mouse models (*Irf1, Nkrf, Pias3, Socs5*) and 12 targets in human cell lines (*BCL2L11, BTG1, CIP2A, FXR1, MEOX2, NKRF, PRKAA1, PTEN, RUNX3, SERPINE1, SMAD4, UVRAG*). Only one target was common to the RA-validated targets in humans and the targets validated in mice, namely the *NKRF*.

**Figure 7:**
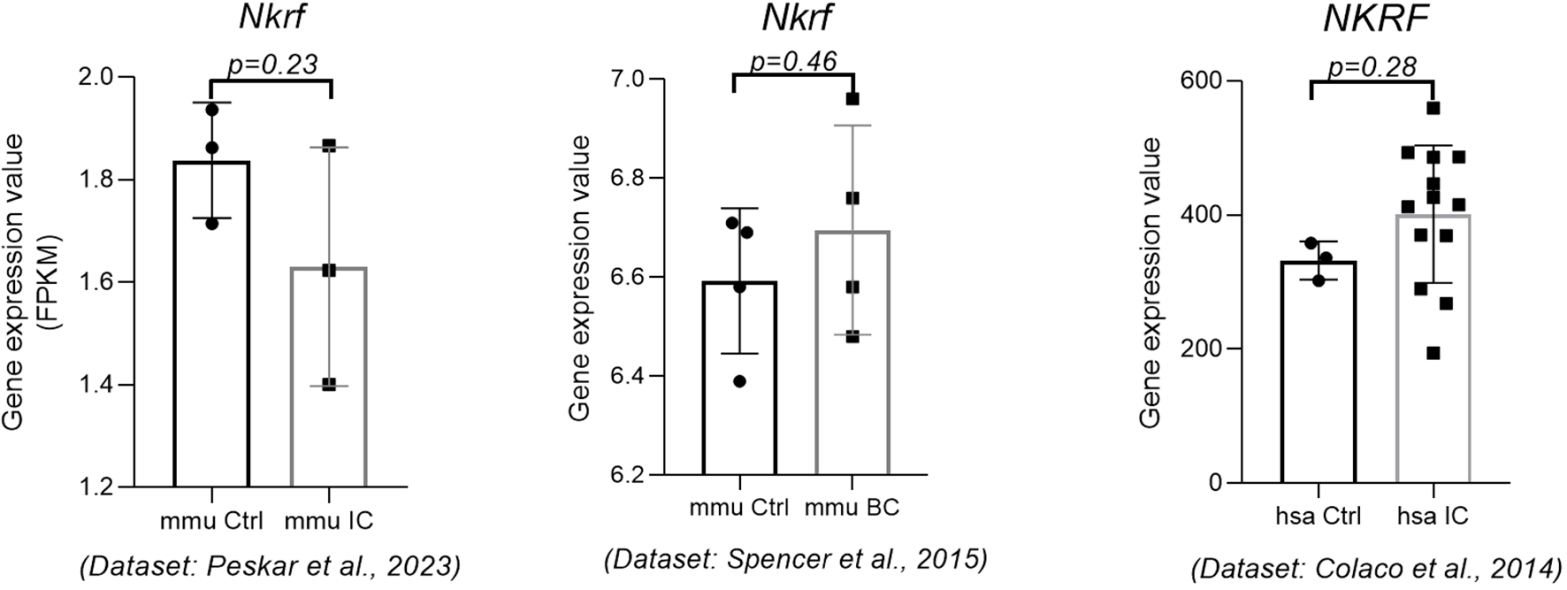
Expression of the *NKRF* gene in mouse IC, mouse BC, and human IC. The expression data were obtained from three independent data sets. p-value was calculated using t-test. Legend: BC, bacterial cystitis; Ctrl, control; IC, interstitial cystitis.

Our results show that the *NKRF* was not significantly deregulated in either BC mice or IC mice (**Figure 7**) (15). Unfortunately, no data on human-derived BC were available at the time of publication. In addition, according to the literature, this gene is also not significantly deregulated in bladder tissue from patients with non-Hunner-type IC (25), accession number GSE57560, and Hunner-type IC (26), accession number GSE11783.

Furthermore, TargetScanMouse was used to predict additional targets for *miR-301a-3p*. Of the predicted targets (799 in total), those with a significant change in expression in bladder tissue of mice with IC compared to CTRL (51 genes with p<0.05 or a fold change of 1.5 or 0.5 in up- or downregulation, respectively (13)) and those with a significant change in expression in bladder tissue of mice with BC (165 genes with p<0.05 (17), **Supplementary Table S5**) were extracted from the mouse datasets. Only 25 genes were deregulated in both mouse groups (49% of genes in the IC group, and 15% of genes in the BC group). The genes that were differentially expressed in the IC bladders were enriched in 6 KEGG pathways, while the genes with differential expression in the BC bladders were enriched in 17 KEGG pathways (**Supplementary Table S5**). Five KEGG pathways were shared by both animal groups.

**Figure 8:**
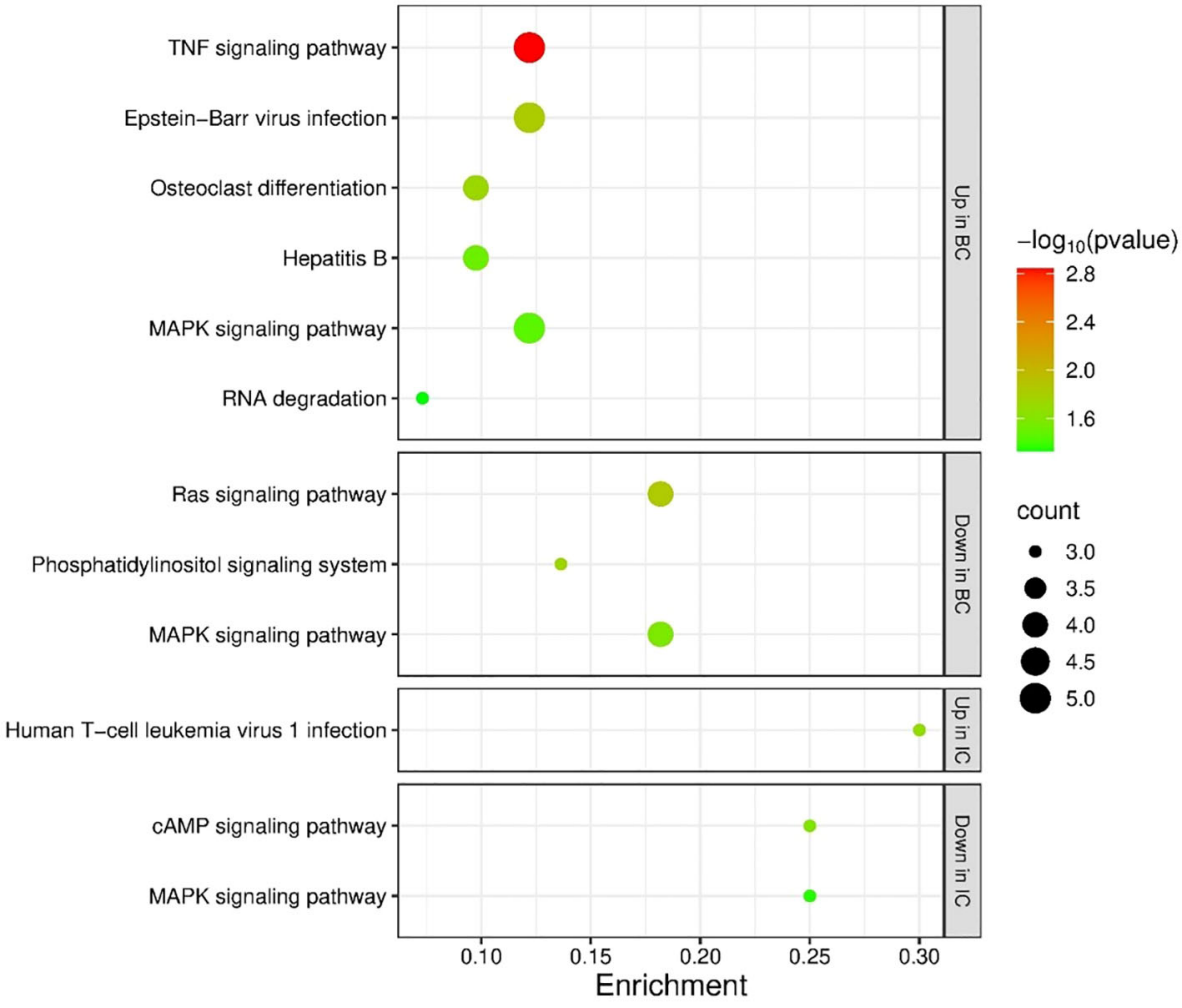
Enrichment analysis of the targets of *miR-301a-3p*. Enrichment was performed for targets with significantly deregulated expression in IC or BC mouse bladders. The figure shows significantly enriched KEGG pathways in all subgroups (targets with upregulated expression in IC or BC, targets with downregulated expression in IC or BC). p-values are represented by the bubble color and gene counts are represented by the bubble size. p-value < 0.05 was considered statistically significant. BC, bacterial cystitis; IC, interstitial cystitis.

As described above, we found that *miR-301a-3p* was inversely regulated between mouse IC and BC bladder samples, specifically upregulated in IC and downregulated in BC, indicating differential regulation of its targets in IC and BC. Of its differentially expressed target genes in IC bladders, 21 genes (41%) were upregulated, and 30 genes (59%) were downregulated, while there was no difference in the number of upregulated (n=82) and downregulated (n=83) genes in BC bladders (**Supplementary Table S5**). The upregulated genes in mouse bladders with BC were enriched in 10 KEGG pathways, while the downregulated genes were enriched in 3 KEGG pathways. ‘MAPK signaling pathway’ was enriched in both subsets of genes in BC. The upregulated genes in mouse bladders with IC were enriched in 1 KEGG pathway, while the downregulated genes were found in 5 KEGG pathways. None of the KEGG pathways were shared by both subgroups of genes in IC. When analyzing the IC data, 1 KEGG pathway was enriched among the upregulated genes, namely ‘Human T-cell leukemia virus 1 infection’, and 1 KEGG pathway was enriched among the downregulated genes, namely ‘MAPK signaling pathway’. The significantly enriched KEGG pathways in BC and IC are shown in **Figure 8**.

## 4 Discussion

In the present study, we aimed to detect the differences in miRNA expression in the urinary bladders of mouse models of IC and BC to identify miRNAs as potential biomarkers to distinguish between these two types of LUTDs. An enrichment analysis of the target mRNAs (of the selected and validated miRNAs) was performed to elucidate the differences in inflammatory mechanisms between the models. In this segment, we found that certain miRNAs could be categorized as inflammation type-independent due to their differential expression in IC and BC compared to controls. On the other hand, we observed miRNAs that were differentially expressed only in BC or IC compared to controls or between BC and IC. We also confirmed that the use of a mouse model of IC is a useful tool to investigate the pathogenic mechanisms of IC in humans as well. This was successfully assessed using the potential target miRNAs selected from publicly available datasets of human patients with IC and validated in mice with IC. We also compared the RA-validated miRNA targets and their expression in humans and mice and found some similarities.

We found that all changes in the expression of miRNAs in BC and IC in comparison to controls had an equal tendency, e.g., upregulation, except *miR-301a-3p*. In addition, the same tendency of expression of 11 miRNAs in human IC and mouse IC and enrichment analysis show a high conservation of biological functions regulated by bladder-expressed miRNAs between human and mouse. The only signaling pathway enriched in mouse bladders (namely the ‘Phagosome’ pathway) may indicate minor species-specific differences in miRNA targeting. However, this strong overlap between species supports the utility of mouse models for studying bladder miRNA functions. Moreover, there are also strong similarities between human and mouse in terms of gene/protein class, localization, and bladder expression of the 8 common genes, supporting the translational relevance of the mouse IC model. However, some of the genes (particularly *HSPB6* and *DLL1*) may have species-specific expression patterns that should be considered when interpreting the extent and mechanisms of inflammation in different species (27).

Our investigation of the histological features and differences between BC and IC in mice revealed that inflammation in the lamina propria is observed in both types of cystitis, especially when considering the number of newly formed vessels and the number of extravascular immune cells. While the number of newly formed blood vessels did not differ statistically significantly between IC and BC, the number of inflammatory cells was significantly higher in BC compared to IC. The number of neutrophils was also significantly higher in BC compared to IC. Clusters of neutrophils were found around the vessels, indicating acute bacterial inflammation. In contrast, there were few neutrophils in the IC animals, with no statistical difference compared to the control mice, confirming chronic inflammation in IC. The results of both analyses were expected and can be explained by the following facts. Namely, acute inflammation is characterized by vasodilation, increased vascular permeability, leukocyte migration to the site of injury, and release of various mediators (28). Neutrophils, as the main innate cellular players in acute inflammation of the urinary tract, eliminate pathogenic bacteria by phagocytosis (29). In general, pro-inflammatory mediators have a short half-life and are rapidly cleared once the noxious stimulus is removed. However, if the stimulus persists, chronic inflammation ensues, as occurs in IC. Excessive infiltration of various cell types can lead to tissue damage and subsequent scarring (e.g., irreversible tissue destruction, fibrosis, and hyperalgesia). All of this is responsible for the chronic waxing and waning of pain and other symptoms of lower urinary tract inflammation (28, 29).

miRNAs that were differentially expressed between BC and control and IC and control, but not between BC and IC, we characterized as inflammation type-independent miRNAs. Only 2 of these miRNAs had previously been associated with IC, namely *miR-320a* and *miR-495* (16, 30, 31). RNA sequencing of miRNA levels in IC tissues and comparison with levels in normal bladder tissue and bladder cancer revealed deregulated expression of 366 miRNAs (203 downregulated and 163 upregulated miRNAs). In particular, the *miR-320* family of miRNAs was downregulated in IC tissue. Genome-wide gene expression analyses and *in silico* database analyses showed that three transcription factors, E2F-1, E2F-2 and TUB, were regulated by *miR-320* family miRNAs. Immunostaining of IC tissues confirmed that these transcription factors are overexpressed in IC tissue (16). Examination of the expression levels of genes involved in the regulation of epithelial permeability, bladder contractility, and inflammation revealed that neurokinin (NK)1 and NK2 tachykinin receptors were significantly downregulated in ICpatients. Among 31 differentially expressed miRNAs in IC patients, a direct correlation between *miR-320* (among others) and downregulation of *NK1R* mRNA and/or protein levels was demonstrated. In the biopsies of IC patients, *miR-320a* was significantly increased (31). Regarding *miR-495*, it has been reported to contribute to the inflammatory response and bladder fibrosis in rats with ulcerative IC via the JAK-STAT signaling pathway by targeting *JAK3*. Namely, after transfection of overexpressed *miR-495* or siRNA-JAK3, a lower extent of mast cell infiltration, reduced number of mast cells, bladder fibrosis, lower NO content, JAK3-positive expression, lower mRNA expression of *JAK3, STAT1, STAT3, TGFβ-1, Col-I, Col-III*, lower protein expression of JAK1, JAK2, JAK3, p-STAT1, p-STAT3, and lower expression of IL-6, IL-8, IL-10, IL-17, and TNF-α were observed. Their findings demonstrate that the overexpression of *miR-495* ameliorates the inflammatory response and bladder fibrosis in ulcerative IC rat models via inactivation of the JAK-STAT signaling pathway through inhibition of JAK3 (30).

We further investigated the expression of miRNAs that were specifically differentially expressed between IC and controls and belong to the inflammation type-dependent miRNAs as we have characterized them. First, we identified two such miRNAs, *miR-34a-5p* and *miR-107* (32, 33). *miR-34a-5p* was the only one previously known to be associated with IC. A study of Vinall et al. (32) was performed to analyze the expression of miRNA in urinary bladder samples obtained from dogs with grossly normal urinary bladder and inflammatory bladder disease. The expression of *miR-34a*, which targets the protein pathways of p53, Rb, or Bcl-2, was higher in the inflammatory bladder disease than in the grossly normal bladders. The findings of this study show that the results of miRNA expression assays can be used to differentiate between samples of grossly normal bladders and bladders from dogs with inflammatory bladder disease (32). Recent evidence suggests that the descending modulatory pathways are important for inflammatory bladder pain. Using animal models and RNAseq analyses, several genes associated with synaptic plasticity (*Grin1, Grip2, Notch1, Arc*, and *Scn2b*) were shown to be upregulated in the cystitis groups compared to controls. *miR-34a-5p* showed cystitis-induced downregulation and bioinformatic analysis identified several 3’UTRs complementary binding sites for *miR-34a-5p* in the *Grin2b, Notch1, Grip2, Scn2b*, and *Arc* genes. It was suggested that long-term molecular alterations play a crucial role in the development of chronic bladder pain as seen in patients with IC (33).

We also observed significant deregulation of inflammation type-dependent miRNAs between BC and control. These included *miR-19a-3p* and *miR-146a* (34–36), which have already been associated with the changes observed in BC. *miR-146a* is one of the key miRNAs that orchestrates immune and inflammatory signaling, often via its recognized target genes, *IRAK1* and *TRAF6*. Its expression is strongly induced by bacterial endotoxin, while its prolonged expression has been associated with immune tolerance, implying that it acts as a fine-tuning mechanism to prevent overstimulation of the inflammatory response. It has also been shown that *miR-146a* plays a role in the control of megakaryocyte and monocyte lineage differentiation and adaptive immunity. *miR-146a* also regulates and mediates signaling through one of the major pattern recognition receptors, the Toll/IL-1 receptors (TLRs) (36). Interestingly, four miRNAs with increased expression were found to play a role in bladder inflammation in IC mice, and one of them was *miR-146a* (35). The authors hypothesize that the mechanism of IC induction may be related to the modulation of specific miRNAs that increase the local and systemic inflammatory response. In contrast to *miR-146a*, there is limited data on the expression of *miR-19a-3p* in the context of the innate immune response and infections. However, Toll-like receptor-7 (TLR7) is functionally involved in the pathogenesis of Hunner-type interstitial cystitis (HIC) in patients. Receiver operating characteristic analysis was used to analyze the diagnostic value of *miR-19a-3p* in patients with HIC, demonstrating inhibited *miR-19a-3p* expression and identifying *TLR7* as a target of *miR-19a-3p* (34). Based on the expression of the RA-validated targets of inflammation type-dependent miRNAs, one could suggest that bacterial infection induces a stronger inflammatory or stress response at the mRNA level. In contrast, induction of sterile bladder inflammation (IC) resulted in less significantly deregulated target mRNAs despite the significant deregulation of miRNAs. The same direction of regulation of target genes (12 out of 16) between the groups might suggests common inflammatory mechanisms between IC and BC. However, the potential regulatory processes might be more subtle in IC, which is also suggested from our histopathological analysis.

In the present study, *miR-301a-3p* was found to be the only miRNA differentially expressed in IC and BC. It has been reported, that *miR-301a*-*3p* is involved in the negative regulation of its target gene for nuclear factor-κB (NF-κB) suppressing factor (*NKRF*), which increases the activation of NF-κB and the production of NF-κB-responsive pro-inflammatory cytokines such as IL-8, IFN-β, NOS2A, and COX-2. The same research showed, that by targeting *NKRF*, *miR-301a-3p* affected the activity of NF-κB and the expression of pro-inflammatory genes downstream of NF-κB in macrophages; this observation may clarify the regulatory role of this miRNA in immune-mediated inflammatory responses (37). Another study reported that HuR, is an RNA-binding protein involved in post-transcriptional regulation that suppresses inflammation and ECM degradation by stabilizing NKRF and inhibiting the NF-κB signaling pathway. A mechanism study showed that HuR promotes *NKRF* mRNA stability and that upregulation of *NKRF* suppresses inflammation and ECM degradation in HuR-deficient cells (38). Our analysis of *miR-301a-3p* expression in IC and BC showed upregulation in IC and downregulation in BC. The proposed mechanism of this miRNA might be trough upregulation of its target *NKRF* in BC and downregulation in IC, leading to increased expression of NF-κB-responsive genes (e.g. IL8) in IC and their downregulation in BC. Pathway enrichment analysis (although not confirmed in our cohort of samples) supported this observation. It is known that NF-κB upregulation is associated with an enhanced recruitment of inflammatory cells and production of pro-inflammatory cytokines at the inflammation site. NF-κB activation and its nuclear localization were detected in bladder biopsies from patients with IC. In the animal model of IC, the strongest nuclear expression of functionally active NF-κB was observed in endothelial cells of bladder microvessels, histiocytes, leukocytes inside and outside blood vessels after CYP administration (28, 29). As for the role of NF-κB in BC, it is reported that the recognition of LPS, a component of the outer membrane of *E. coli*, by urothelial cells requires a number of proteins, including CD14, which is an important factor in IL-8 induction in the bladder urothelial response to LPS. It was found that after LPS treatment in the animal model of BC, NF-κB is rapidly localized in the nuclei of urothelial cells, peaking after 30–60 minutes and returning to control levels after 24 hours (39). Other studies suggest that the urothelium releases several neuropeptides (e.g., nerve growth factor) and neurotransmitters that activate submucosal afferent nerves and mast cells, resulting in hyperalgesia in patients with IC. Mast cells are activated by numerous mediators such as cytokine-like stem cell factors and nerve growth factors released from the damaged urothelium, bacterial and viral antigens, neuropeptides, neurotensin, etc. Once activated, mast cells release leukotriens such as IL-6 and IL-8, and others. IL-8 is a known gene suppressed by NKRF, which is downregulated in IC. IL-8 is overexpressed in more than 50% of IC bladders (28, 29).

Finally, we should recognize that IC is a highly complex human disorder with unknown etiology and poorly understood pathophysiology, so the translational relevance of animal models of IC must be interpreted with caution. Multiple animal models of IC have been developed using different induction strategies, as comprehensively reviewed by Birder and Andersson (40). Among these, the CYP-induced model used in the present study is the most widely used and reported bladder-centric model, as it reliably reproduces several key functional and histological features of the human IC. However, it is clear that no single experimental model, regardless of induction method or animal type, can fully capture the multifaceted nature and clinical heterogeneity of symptoms experienced by patients with IC.

The main disadvantage of our study is the small sample size, and independent validation in larger cohorts is essential to confirm the proposed BC- and IC-specific miRNA signature. However, the advantage of this work is the translation of identified miRNAs from humans to a mouse model, which could be a useful tool for investigating IC. However, the study would also benefit from validation of the identified RA-validated targets in bladder cell line models and by protein staining in bladder tissue of IC and BC.

In summary, the mouse model of IC shares some common features with human IC, suggesting that it is a useful tool for identifying new biomarkers and therapeutic targets for IC, as well as for developing new diagnostic approaches when other diagnostic methods fail. We identified *miR-301a-3p* as a molecular marker that may discriminate between two types of cystitis, as it is differentially expressed in IC and BC. However, further validation with a larger mouse sample size and human biopsies is needed for confirmation. In addition, we found upregulation of *NKRF* and NF-κB as a common feature of IC in both humans and mice, suggesting that both may serve as potential therapeutic targets in the future.

## Data Availability

The original contributions presented in the study are included in the article/**Supplementary Material**. Any further inquiries can be directed to the corresponding author. The original contributions presented in the study are publicly available. This data can be found here: NCBI GEO (accession number GSE221783).
