# Supplementary material for: miRNA expression profile as a potential tool for discrimination between bacterial and interstitial cystitis
Source: Front Immunol. 2026 Feb 16;17:1738839. doi: 10.3389/fimmu.2026.1738839 (PMC12950535; doi:10.3389/fimmu.2026.1738839)
Supplement: Supplementary file 1 [file Table1.docx]

Supplementary Material

# Supplementary Tables

**Supplementary table S1: Human RA-validated targets.** miRTarBase-derived data of targets of all 20 miRNAs that were significantly deregulated in mouse bladder FFPE samples. The data represents reporter assay-validated targets in human tissue.

**Supplementary table S2**: **Mouse RA-validate targets.** miRTarBase-derived data of targets of 20 miRNAs that were significantly deregulated in mouse bladder FFPE samples. The data represents reporter assay-validated targets in mouse tissue. RA-validated targets were found for 16 out of 20 miRNAs.

**Supplementary Table S3:** **Comparison of mouse and human data**. List 1: RA-validated targets of miRNAs with significant deregulation between IC and Ctrl mouse bladder FFPE samples. A list of targets expressed in mouse and human bladders is provided. List 2: Functional annotation of bladder-expressed targets. Enrichment of the targets in KEGG pathways is presented. List 3: Human protein atlas and MGI-derived data for common RA-confirmed targets in mouse and human of differentially expressed miRNAs between IC and CTRL groups. A summary table is also presented. List 4: Expression data of the common targets in mouse and human IC relative to controls.

**Supplementary table S4: Targets of inflammation type-dependent and type-independent miRNAs.** List 1: RA-validated targets of inflammation type-dependent miRNAs. P values and fold change of target expression in IC and BC mouse models are presented. List 2: RA-validated targets of inflammation type-independent miRNAs. P values and fold change of target expression in IC and BC mouse models are presented. List 3: Functional annotation of targets of inflammation type-dependent and -independent miRNAs. Enrichment of the targets in KEGG pathways is presented.

**Supplementary table S5: *miR-301a-3p* data**. List 1: *miR-301a-3p* targets predicted with TargetScanMouse, and targets significantly deregulated in mouse IC and mouse BC.
